# Mechanical Properties, Constitutive Behaviors and Failure Criteria of Al-PTFE-W Reactive Materials with Broad Density

**DOI:** 10.3390/ma15155167

**Published:** 2022-07-26

**Authors:** Tao Sun, Aoxin Liu, Chao Ge, Ying Yuan, Haifu Wang

**Affiliations:** Beijing Institute of Technology, 5 South Zhongguancun Street, Beijing 100081, China; 3120185143@bit.edu.cn (T.S.); 1120183024@bit.edu.cn (A.L.); gechao@bit.edu.cn (C.G.); 3120185181@bit.edu.cn (Y.Y.)

**Keywords:** reactive materials, Al-PTFE-W composites, tension failure, mechanical properties, Johnson–Cook modeling

## Abstract

Quasi-static tension tests, quasi-static compression tests and dynamic compression tests were conducted to investigate the mechanical properties, constitutive behaviors and failure criteria of aluminum-polytetrafluoroethylene-tungsten (Al-PTFE-W) reactive materials with W content from 20% to 80%. The analysis of the quasi-static test results indicated that the strength of the materials may be independent of the stress state and W content. However, the compression plasticity of the materials is significantly superior to its tension plasticity. W content has no obvious influence on the compression plasticity, while tension plasticity is extremely sensitive to W content. Dynamic compression test results demonstrated the strain rate strengthening effect and the thermal softening effect of the materials, yet the dynamic compression strengths and the strain rate sensitivities of the materials with different W content show no obvious difference. Based on the experimental results and numerical iteration, the Johnson–Cook constitutive (*A*, *B*, *n*, *C* and *m*) and failure parameters (*D*_1_~*D*_5_) were well determined. The research results will be useful for the numerical studies, design and application of reactive materials.

## 1. Introduction

Reactive materials, which not only meet a certain mechanical strength but also have chemical energy release characteristics, are classified as a kind of energetic structural material [1]. Different from metals or explosives, such material remains inert under ambient conditions but reacts violently with a large amount of chemical energy release under dynamic loading. The materials have potential applications in aerospace, fortifications and efficient damage. For example, the shield structures made of reactive materials defend against space debris more effectively than those made of inert materials [2]. Additionally, the reactive material projectile shows a combined damage of kinetic energy and chemical energy when it impacts an aluminum plate, and the average diameter of perforation is 4–19 times the diameter of the projectile [3].

Al-PTFE composites are a typical representative of reactive materials. Much research has been conducted on fabrications, mechanical property, constitutive behavior and energy release characteristics of Al-PTFE reactive materials. Joshi [4] patented a preparation process of Al-PTFE reactive materials, which mainly includes powder mixing, molding and sintering. The samples fabricated by the method can withstand a larger overload without breaking. Based on quasi-static and dynamic compression tests, Ge [5] studied the mechanical property of Al-PTFE (26.5 wt. %/73.5 wt. %) reactive materials. The results demonstrated that the materials show typical elasto-plastic behavior with prominent strain hardening, strain rate strengthening and thermal softening effect. Raftenberg [6] used the Johnson–Cook constitutive parameters and simulated the impact deformation of Al-PTFE rods. Wang [7] found a mechanical property transforming response from brittle to ductile by comparing the effects of sintering temperature, cooling rate and initial and final cooling temperature on the properties of Al-PTFE reactive materials. Through numerical simulation, Tang [8] found that with Al content increasing, the ultimate compressive strength of Al-PTFE reactive materials increased first and then decreased, and failure mode evolved from the shear failure of matrix to debonding failure of particles. Feng [9,10] found that Al-PTFE composites would react under quasi-static compression when fabricated at a pressure of 60 MPa and a sintering temperature of 350 °C; he then proposed a crack-induced initiation mechanism. Osborne [11] revealed the thermal reaction mechanism of Al-PTFE reactive materials. Mock and Holt [12] investigated the shock initiation of Al-PTFE rods using a gas gun and then obtained the relationship between the initiation pressure threshold and initiation time. Ames [13] proposed a vented chamber calorimetry for measuring and evaluating the impact energy release; the measured blast pressure reached almost 110 psig.

Higher density, greater strength, more reaction energy and greater insensitivity of reactive materials are expected for engineering applications. Although Al-PTFE composites have high energy release rates, their density and strength are relatively low compared to metals such as steel or tungsten, resulting in poor kinetic energy and penetration ability. At present, one of the most effective methods is adding high-density metals (e.g., tungsten) into the mixture of Al and PTFE powders. The density of Al-PTFE-W reactive materials can be improved to steel-like density (7.8 g/cm^3^) or even higher by controlling W content. Xu [14] reported that the average yield stress of Al-PTFE-W reactive materials, fabricated at a pressure of 200 MPa and a sintering temperature of 380 °C, increased from 9.2 MPa to 23.5 MPa as the density increased from 2.61 g/cm^3^ to 9.28 g/cm^3^ under quasi-static compression. In addition, he found that the sintered Al-PTFE-W reactive materials showed higher strength and greater fracture toughness compared with the pressed-only materials. Herbold [15] demonstrated that materials with fine W particles have a higher ultimate compressive strength compared to those with coarse W particles. Cai [16] revealed that the failure of Al-PTFE-W reactive materials was concentrated primarily in the PTFE matrix, and that the debonding between W particles and the matrix provided the initiation and propagation of cracks. Zhou [17] found that W content influences the trend of reaction efficiency by affecting the shock temperature and the initial shock pressure of Al-PTFE-W reactive materials. Ren [18] revealed that the impact reaction process of Al-PTFE-W reactive materials includes deformation, failure and combustion reaction. Moreover, she found that the reaction threshold of the materials increases with the increase in W content. However, Ge [19] obtained an opposite conclusion, in which the impact-initiation sensitivity increased with the increased W content when the materials were fabricated at a much lower sintering temperature and shorter duration. Xu [20,21] conducted ballistic experiments and indicated that the damage of the aluminum plate impacted by an Al-PTFE-W reactive material projectile was determined directly by the mechanical strength and fragmentation degree of the materials. When describing the mechanical behavior of materials subjected to significant strain, a high stain rate and temperature, constitutive model and failure criteria play an important role in simulating the deformation and fragmentation of materials under different loadings, such as impact and blast. Zhang [22] obtained Johnson–Cook constitutive parameters of Al-PTFE-W reactive materials with W content from 0% to 50% based on quasi-static and dynamic compression tests. However, the dynamic mechanical properties and constitutive behaviors of Al-PTFE-W reactive materials with higher density are rarely reported. Moreover, the failure criteria of Al-PTFE-W reactive materials remain to be explored.

For this paper, quasi-static compression tests, quasi-static tension tests and dynamic compression tests were conducted to investigate the mechanical properties, constitutive behaviors and failure criteria of Al-PTFE-W reactive materials with W content from 20% to 80%. The effects of W content, stress state, strain rate and temperature on the mechanical properties of the materials were analyzed and discussed. Finally, based on the experimental results and numerical iteration, Johnson–Cook constitutive parameters and failure parameters were obtained.

## 2. Experimental

### 2.1. Specimen Fabrication

**Figure 1:**
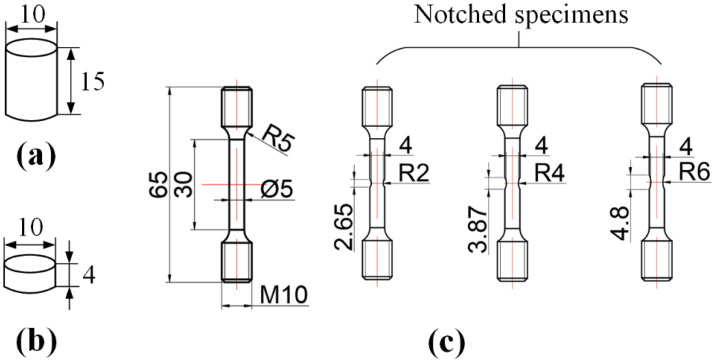
Schematics of specimens prepared: (**a**) quasi-static compression specimens, (**b**) dynamic compression specimens and (**c**) quasi-static tensile specimens.

**Figure 2:**
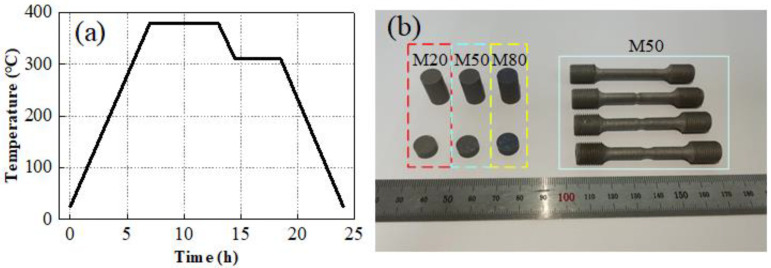
(**a**) Temperature history of sintering cycle and (**b**) typical specimens.

In this work, three different types of specimens, i.e., quasi-static compression specimens, quasi-static tensile specimens and dynamic compression specimens, were prepared to investigate the constitutive behaviors and failure criteria of Al-PTFE-W reactive materials. All specimens were designed based on the Chinese standard GB/T 7314-2017 and GB/T 228.1-2010, as shown in Figure 1. *R* is the notched radium. The preparation of the specimens mainly includes component mixing, molding and vacuum sintering. Firstly, powders of PTFE (100 nm), Al (24 μm) and W (44 μm) with a certain mass ratio were mixed uniformly by a V-shaped mild machine (Zhongcheng Pharmacy Machine Co., Ltd., Hunan, China, VH-50) for 40 min. Keeping the mass ratio between PTFE and Al at about 73.5:26.5, Al-PTFE-W reactive materials with three different W contents were prepared in this research, as tabulated in Table 1. TMD is the theoretical maximum density. After this, the uniform mixtures were isostatically pressed at a pressure of 200 MPa in a self-designed steel model with an inner diameter of 10 mm. Finally, the pressed samples were sintered in an oven filled with argon. The temperature history of the sintering cycle is shown in Figure 2a. It is noted that the specimen expands slightly after sintering. The actual density is generally lower than the theoretical maximum density. For the tensile samples in particular, additional machining was required to form the smooth round bar specimens and the notched specimens. Typical specimens are presented in Figure 2b.

### 2.2. Quasi-Static and Dynamic Test Methods

1. Quasi-static compression and tension

The quasi-static compression and tension tests were conducted using the MTS universal material testing machine. For the quasi-static compression specimens and the smooth bound bar specimens, the strain rate, the true stress and the true strain can be expressed as

(1) $${\dot{\varepsilon }}_{s}(t)=\frac{v(t)}{l_{0}}$$

(2) $$\sigma_{s}(t)=\left(1\pm \frac{\Delta l}{l_{0}}\right)\frac{F(t)}{A_{0}}$$

(3) $$\varepsilon_{s}=\pm \ln\left(1\pm \frac{\Delta l}{l_{0}}\right)$$

where ${\dot{\varepsilon }}_{s}(t)$ is the strain rate, *v*(*t*) is the loading speed, *l*_0_ is the effective length of the specimen and *σ*_s_(*t*) and *ε*_s_(*t*) are the true stress and the true strain, respectively. *F*(*t*) is the loading force, *A*_0_ is the initial cross-sectional area of the specimen. ‘+’ and ‘−’ correspond to tension and compression, respectively. To correspond to an equal strain rate of 0.001 s^−1^, the loading speeds *v*(*t*) were set to 0.9 mm/min and 1.8 mm/min for compression and tensile condition, respectively.

2. Dynamic compression at elevated temperatures

**Figure 3:**
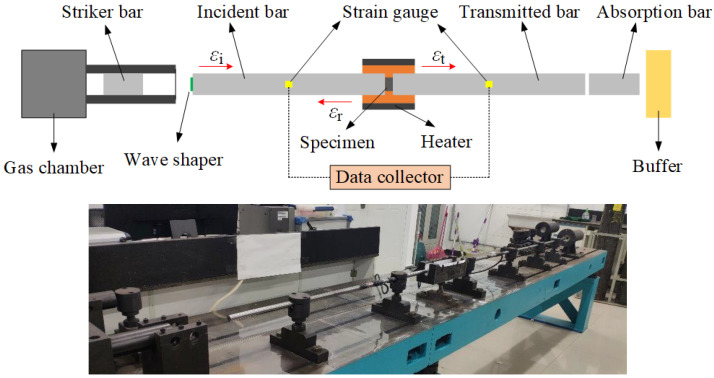
The SHPB system.

The dynamic compression tests were performed using the Split Hopkinson Pressure Bar (SHPB) system equipped with 16 mm diameter 7075 T6 aluminum bars, as shown in Figure 3. Prior to the experiment, the specimen is set between the incidence bar and the transmitted bar. The striker bar is driven by the gas chamber to impact the incidence bar, which results in a compression stress wave that propagates through the bar and provides impact loading on the specimen. The impact velocity can be controlled by the gas chamber. When the compression stress wave propagates to the interface of the incidence bar and the specimen, the reflected wave and the transmitted wave are formed and then propagate toward the incidence bar and the transmitted bar, respectively. The strain gauges glued on the bars record the strain signals. Combined with the one-dimensional stress wave theory and the strain signals, the true stress–strain curves of the specimen can be calculated [5], as presented by Equations (4)–(6):

(4) $${\dot{\varepsilon }}_{d}(t)=\frac{C_{0}}{l_{0}}\left[\varepsilon_{i}(t)-\varepsilon_{r}(t)-\varepsilon_{t}(t)\right]$$

(5) $$\sigma_{d}(t)=\frac{E_{b}A_{b}}{2A_{0}}\left[\varepsilon_{i}(t)+\varepsilon_{r}(t)+\varepsilon_{t}(t)\right]$$

(6) $$\varepsilon_{d}(t)=-\frac{C_{0}}{l_{0}}\int_{0}^{t}\left[\varepsilon_{i}(t)-\varepsilon_{r}(t)-\varepsilon_{t}(t)\right]dt$$

where ${\dot{\varepsilon }}_{d}(t)$ is the strain rate and *C*_0_ is the sound speed in the bar. *ε*_i_(*t*), *ε*_r_(*t*) and *ε*_t_(*t*) are the incidence, reflected and transmitted strain pulse, respectively. *σ*_d_(*t*) and *ε*_d_(*t*) are true stress and strain of the specimen, respectively. *E*_b_ and *A*_b_ are the Young’s modulus and the cross-sectional area of the bar, respectively. Note that the dynamic loading of the specimens at elevated temperatures requires the employment of a furnace. The temperature of the furnace was set to 25 °C, 100 °C, 150 °C and 200 °C, respectively. 

## 3. Results and Discussion

### 3.1. Stress State Analysis under Quasi-Static Condition

**Figure 4:**
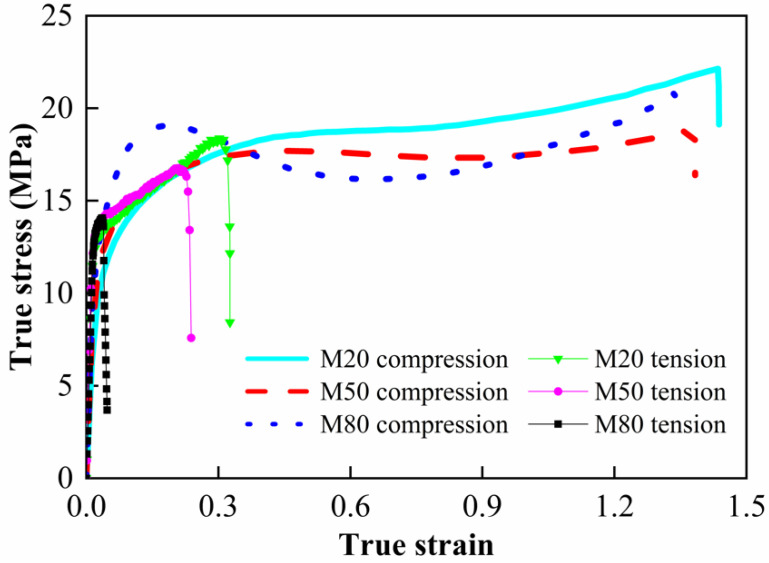
True stress–strain curves in quasi-static tests.

The true stress–strain curves of quasi-static compression and smooth round bar specimens are shown in Figure 4. From the stress–strain curves, typical elastic–plastic properties could be observed. When the ultimate strength is attained, the materials fail and the stress drops rapidly. Relevant material characteristic parameters were obtained according to the curves, as listed in Table 2. Herein, the elastic modulus is defined as the slope of the straight elastic stage. For the curves with obvious yield process, the yield strength is defined by the lower yield point. For the curves without obvious yield, the yield strength is determined by a 0.2% plastic offset [5,22].

From Table 2, it is found that the ultimate strength under compression is slightly higher than that under tension, while the critical failure strain under compression is significantly higher than that under tension. The elastic modulus and the yield strength both show an increasing tendency with the increased W content, indicating the W particle strengthening effect on the deformation resistance of the materials. However, the critical failure strain shows a decreasing trend with the increased W content. Generally, the failure modes of the materials mainly include the shear failure of matrix and the debonding failure of particles [8]. The increased W particles break the continuity of the PTFE matrix and accelerate the formation of cracks, thus resulting in the premature failure of the materials. It should be noted that as the W content increases from 20% to 80%, the critical failure strain decreases by 6.2% and 88% under compression and tension, respectively. The significant difference indicates that the materials’ failure is more sensitive to W content under tension than under compression. This may be attributed to force chain effect [8]. Under compression, the metal particles are linked with each other from the top to the bottom in the matrix, forming several force chains which bear and transmit the main compressive load. The force chains effectively prevent further deformation of the PTFE matrix. Furthermore, the force chain effect is enhanced as W content increases. However, no force chain is formed when the materials are tensioned. The addition of W particles only accelerates the materials’ failure.

**Figure 5:**
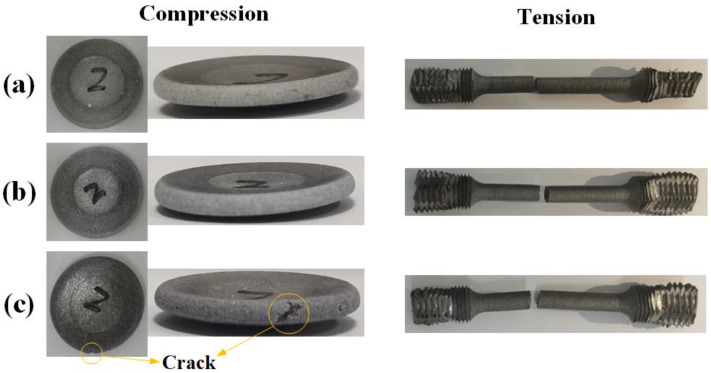
Samples after quasi-static loading. (**a**) M20, (**b**) M50 and (**c**) M80.

Figure 5 presents the samples after quasi-static loading. For the smooth round bar specimens, no obvious necking is observed, while the compressed specimens assume the shape of a round cake. An obvious crack appears in the M80, indicating again that the increased W particles break the continuity of the PTFE matrix. Furthermore, this may provide a failure mechanism induced by internal damage accumulation. It should be noted that in the quasi-static tensile test, both ends of the sample are connected to the MTS universal material testing machine by fixtures. Due to the low strength of the materials, the clamped thread section seriously deforms after the test.

**Figure 6:**
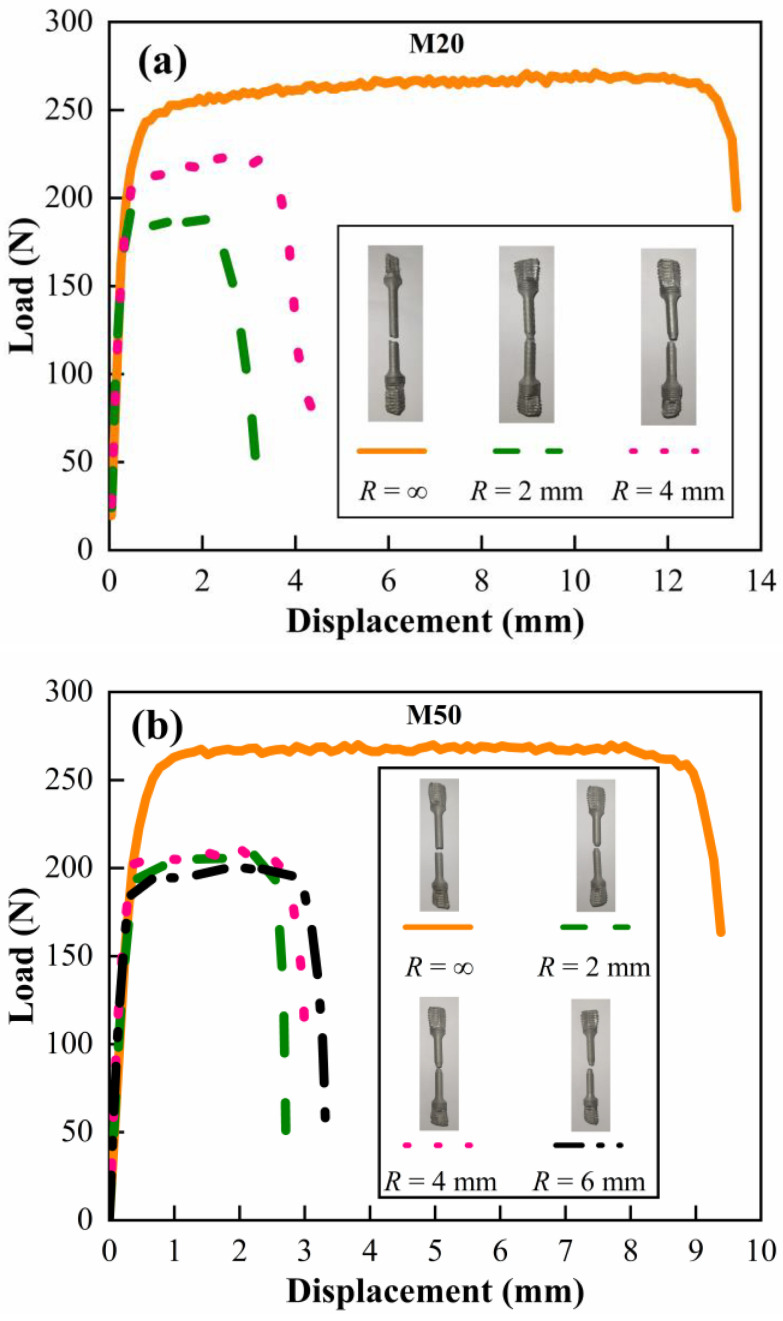

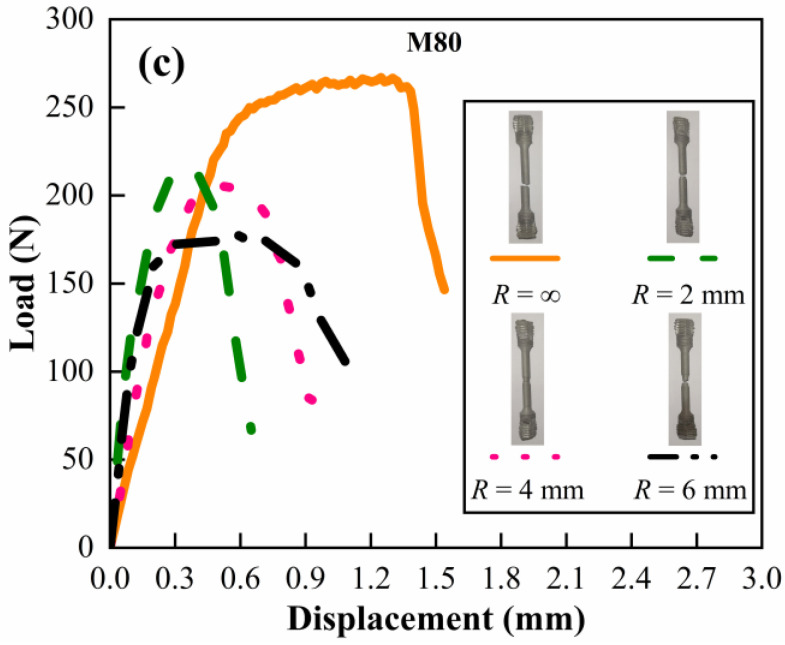
Load–displacement curves in quasi-static tensile tests for (**a**) M20, (**b**) M50 and (**c**) M80.

The load–displacement curves of smooth and notched bound bar specimens under quasi-static tension, as well as the photographs of the specimens after loading, are shown in Figure 6. The smooth bound bar is regarded as *R* = ∞. It is evident in Figure 6 that the elongation at break increases with the increased notch radius, which is consistent with the properties of metal materials [23]. In addition, it is found in Figure 6 that the elongation at break of the notched specimen decreases with the increased W content, which is consistent with the variation in the smooth bound bar specimen.

**Figure 7:**
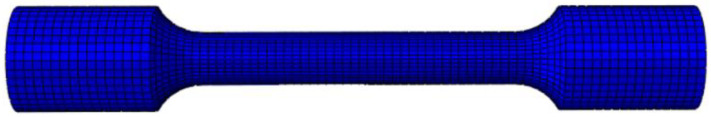
Numerical model of smooth bound bar specimen.

**Figure 8:**
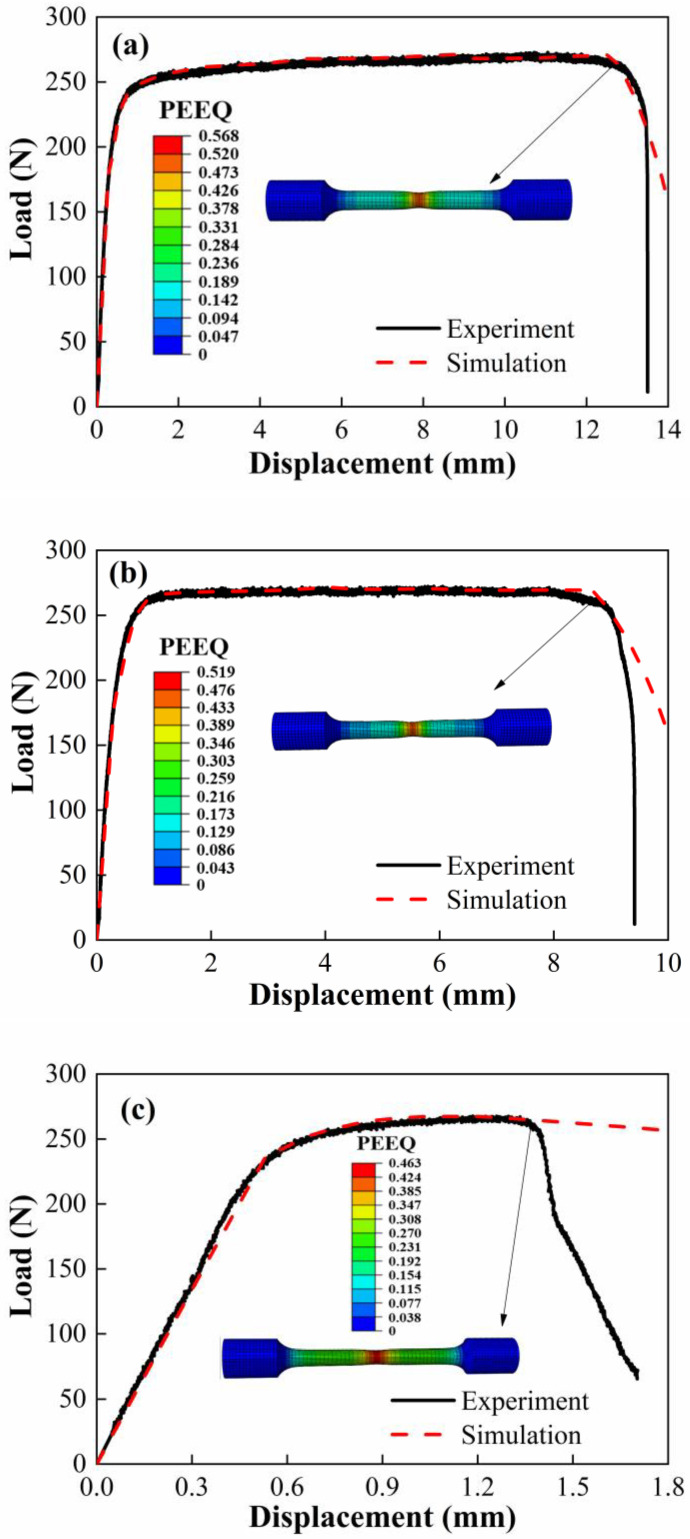
Equivalent plastic strain cloud diagram of smooth round bar sample at fracture time. (**a**) M20, (**b**) M50 and (**c**) M80.

In order to obtain the fracture strain of tensile specimens, the method described in reference [24] was adopted. The method is thus briefly described here. Firstly, the finite element software Abaqus/Standard is used to establish the numerical model of the tensile specimen. Then, all elements on the center section of the sample are taken as the research object, and the simulated load–displacement curve is modified through iteration until it corresponds to the experimental load–displacement curve. Finally, when the simulated load–displacement curve reaches the experimental fracture point, the simulated equivalent plastic strain is regarded as the fracture strain of the specimen. Figure 7 and Figure 8 show the numerical model and the equivalent plastic strain cloud diagram at the fracture time of the smooth bound bar specimen, respectively. It is observed that the simulated curves are in good agreement with the experimental curves before specimen fractures. Furthermore, the stress triaxiality σ* was also obtained by the simulation. The stress triaxiality σ* is defined as the ratio of hydrostatic pressure *σ*_m_ to von Mises equivalent stress *σ*_eq_, which can be expressed as

(7) $$\sigma *=\frac{\sigma_{m}}{\sigma_{\mathrm{eq}}}=\frac{\sigma_{11}+\sigma_{22}+\sigma_{33}}{3\sigma_{\mathrm{eq}}}$$

where *σ*_11_, *σ*_22_ and *σ*_33_ are the three normal stresses.

**Figure 9:**
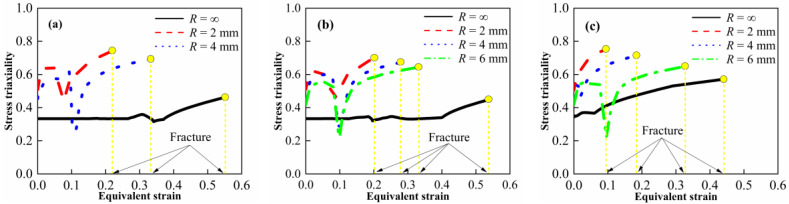
Stress triaxiality varies with equivalent strain for (**a**) M20, (**b**) M50 and (**c**) M80.

**Figure 10:**
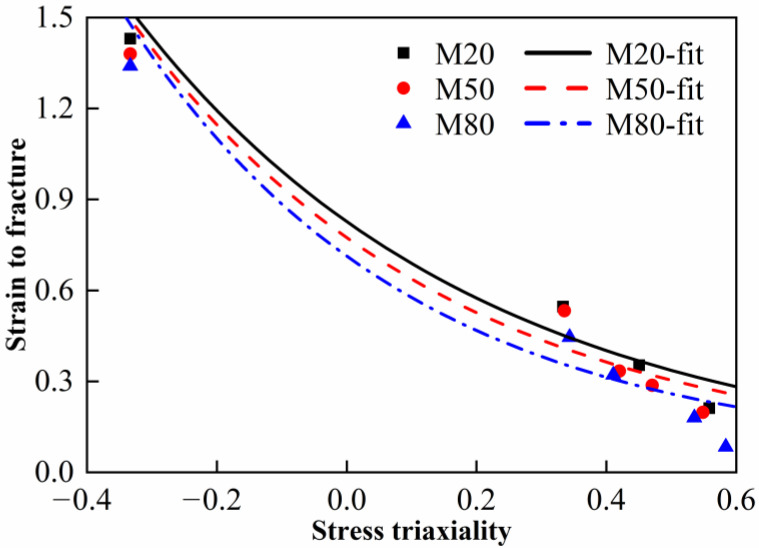
Strain to fracture versus the stress triaxiality.

According to the simulations and Equation (7), the relationship between the stress triaxiality, the equivalent plastic strain and the fracture strain was obtained, as shown in Figure 9. The stress triaxiality shows an increasing trend upon continuous loading. The sudden drop in stress triaxiality may be attributed to the transition from elasticity to plasticity. The stress triaxiality gradually increases with the decrease in notch radius, which is consistent with the properties of metal materials. To describe the stress triaxiality and the strain to fracture quantitatively, the average value of stress triaxiality from the initial time to fracture was calculated. Furthermore, the quasi-static compressive experimental data were added and its corresponding stress triaxiality is about −1/3 [25], as shown in Figure 10.

### 3.2. Strain Rate and Thermal Effect under Dynamic Compression

**Figure 11:**
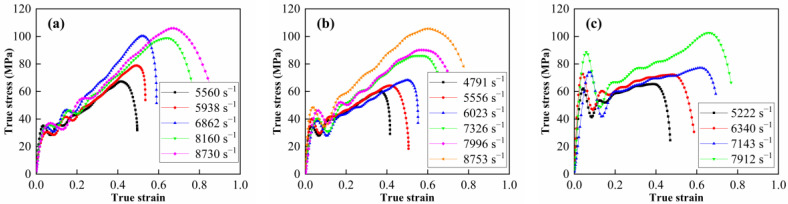
True stress–strain curves of Al-PTFE-W materials at elevated strain rates at 25 °C. (**a**) M20, (**b**) M50 and (**c**) M80.

**Figure 12:**
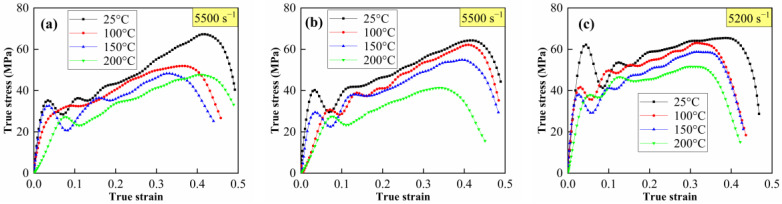
True stress–strain curves of Al-PTFE-W materials at elevated temperatures. (**a**) M20, (**b**) M50 and (**c**) M80.

**Figure 13:**
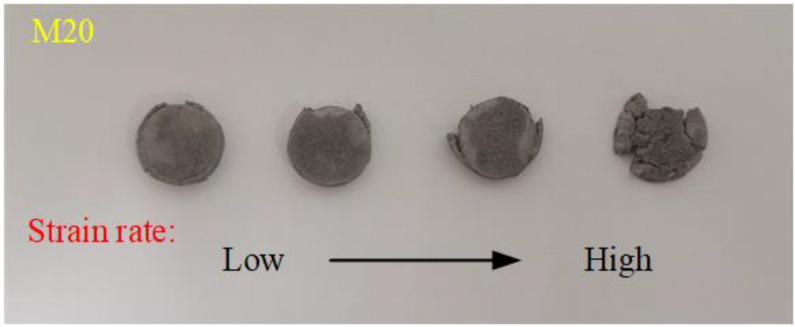
Typical recycled residues after dynamic compression.

The true stress–strain curves of the Al-PTFE-W materials at elevated strain rates and temperatures are shown in Figure 11 and Figure 12, respectively. Note that the curves in Figure 12 are approximately the same at elevated temperatures, except for M20 at 100 °C. This may be because the specimen does not fit tightly with the incidence bar and the transmission bar, which affects the wave propagation. Similar to quasi-static compression, the materials also exhibit typical elastic–plastic properties under dynamic compression. Compared to quasi-static compression, obvious yield behavior and more significant strain hardening effect can be observed from the dynamic compression curves. In particular, the yield strength under dynamic compression is about 2 to 3 times that under quasi-static compression. All of the specimens’ tested fractures and typical recycled residues are presented in Figure 13. Clearly, the materials fracture more seriously with increased strain rate. Relevant material characteristic parameters obtained from Figure 11 and Figure 12 are listed in Table 3 and Table 4. Herein, the yield strength is determined by the lower yield point. From Table 3, the yield strength, the ultimate strength and the critical failure strain all show an increasing trend with the elevated strain rate, demonstrating the strain rate strengthening effect. In contrast, the material characteristic parameters in Table 4 all present a decreasing trend with the elevated temperature, indicating the thermal softening effect. On the one hand, the strength of each component in the materials decreases at elevated temperatures. On the other hand, the elevated temperature decreases the viscosity between the matrix and metal particles. As a result, the debonding failure is facilitated by elevated temperatures.

**Figure 14:**
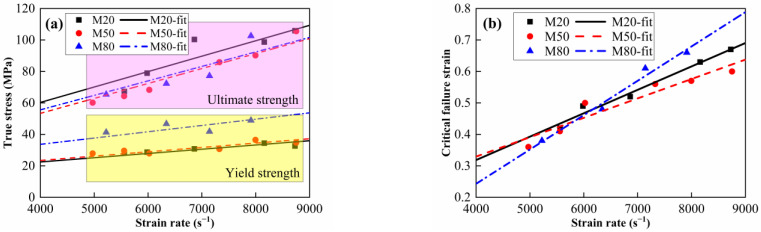
Strain rate effect on yield strength, ultimate strength and critical failure strain of the material with different W contents. (**a**) Strength and (**b**) critical failure strain.

To study the effect of W content on strain rate sensitivity, the data in Table 3 were linearly fitted, as shown in Figure 14. It is observed from Figure 14a that with the increase in W content, the yield strength gradually increases while the ultimate strength decreases first and then increases, which is consistent with the quasi-static compression characteristics. The linear slope of ultimate strength proves to be close to the strain rate for the materials with different W contents. The linear slope of yield strength with respect to strain rate shows a slightly increasing trend with increasing W content, indicating that the sensitivity of the materials to strain rate increases slightly with increasing W content. This should be attributed to the dislocation density difference between the deformed and undeformed zone. However, the critical failure strain shows a complex trend in the tested strain rate range. When the strain rate is less than 6000 s^−1^, the variation in the critical failure strain with W content is essentially similar to that under quasi-static compression. When the strain rate is higher than 6000 s^−1^, the critical failure strain follows the following order: M80 > M20 > M50, and the difference between them becomes more significant with the increasing strain rate, as shown in Figure 14b. This may be attributed to the temperature rise during plastic deformation. Most of the plastic work is converted to heat under high strain rate loading [6], which significantly reduces the matrix strength. As the strain rate increases, the matrix softening effect becomes more obvious. As a result, the force chain strengthening effect dominates. Relevant studies have indicated that the bearing capacity of force chains is not only dependent on the number of metal particles, but also on the number and spatial distribution of force chains [26]. Furthermore, the strength of the metal particles also decreases due to the temperature rise. Therefore, the force chain strengthening effect may not be enough to compensate for the softened matrix when W content is less than 50%.

### 3.3. Constitutive and Failure Modeling

The Johnson–Cook model, which includes a constitutive model and failure model, was applied to describe the constitutive behavior and failure criteria of the materials [27]. The Johnson–Cook constitutive model expresses the equivalent flow stress as a function of the equivalent plastic strain, the strain rate and the temperature, which can be written as

(8) $$\sigma =\left[A+B{\left(\varepsilon_{p}\right)}^{n}\right]\left[1+C\ln\left(\frac{\dot{\varepsilon }}{{\dot{\varepsilon }}_{0}}\right)\right]\left[1-{\left(\frac{T-T_{r}}{T_{m}-T_{r}}\right)}^{m}\right]$$

where *σ* is the equivalent stress, *ε*_p_ is the effective plastic strain, $\dot{\varepsilon }$ is the effective strain rate, ${\dot{\varepsilon }}_{0}$ is the reference strain rate (0.001 s^−1^), *T* is the absolute temperature, *T*_r_ is the reference temperature (25 °C), *T*_m_ is the melting temperature of the materials (600 °C) and *A*, *B*, *C*, *n* and *m* are material constants. Based on the method described in reference [5], the compressive constitutive parameters (*A*, *B*, *n*, *C* and *m*) for the materials are obtained, as listed in Table 5.

**Figure 15:**
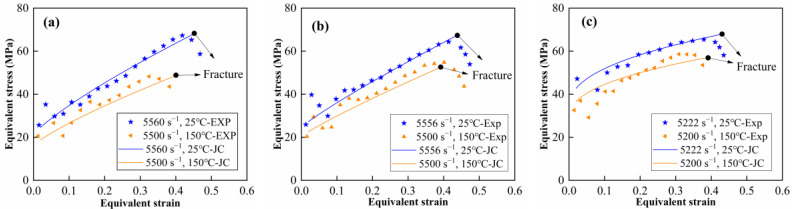
Comparison between the tested and JC model predicted stress–strain curves: (**a**) M20, (**b**) M50 and (**c**) M80.

Based on the damage accumulation during the deformation, the Johnson–Cook failure model presents the strain to fracture as a function of the stress triaxiality, the strain rate and the temperature. The damage to an element is defined as

(9) $$D=\sum \frac{\Delta \varepsilon_{p}}{\varepsilon_{f}}$$

where *D* is the damage parameter and fracture is then allowed to occur when *D* = 1.0. Δ*ε*_p_ is the increment of equivalent plastic strain and *ε*_f_ is the equivalent strain to fracture, which can be expressed as

(10) $$\varepsilon_{f}=\left[D_{1}+D_{2}\exp\left(D_{3}\sigma *\right)\right]\left[1+D_{4}\ln\left(\frac{\dot{\varepsilon }}{{\dot{\varepsilon }}_{0}}\right)\right]\left[1+D_{5}\left(\frac{T-T_{r}}{T_{m}-T_{r}}\right)\right]$$

where *D*_1_~*D*_5_ are five material constants and σ^*^ is the stress triaxiality. The constants *D*_1_, *D*_2_ and *D*_3_ were determined by the fitted curves in Figure 10. The constants *D*_4_ and *D*_5_ were determined by dynamic compression tests at various strain rates and temperatures, respectively. Note that the critical failure strain under dynamic compression is approximately regarded as the strain to fracture in this paper. The Johnson–Cook failure parameters for the materials are listed in Table 6. Comparison between the experimental data and the JC model is shown in Figure 15. It can be observed that a good agreement is achieved between the predicted and experimental stress–strain curves. The Johnson–Cook model obtained in this paper can provide effective help to the numerical studies of the materials.

## 4. Conclusions

Quasi-static compression tests, quasi-static tension tests and dynamic compression tests were conducted to investigate the mechanical properties, constitutive behaviors and failure criteria of Al-PTFE-W reactive materials with W content from 20% to 80%. The following conclusions were drawn:

(1) Under quasi-static (10^−3^ s^−1^) condition, the strength of the materials may be independent of stress state and W content. Regardless of compression or tension, the strength of the materials with W content from 20% to 80% ranges from 10 MPa to 20 MPa.
(2) Under quasi-static condition, the compression plasticity of the materials is significantly superior to its tension plasticity. W content has no obvious influence on the compression plasticity, while tension plasticity is extremely sensitive to W content. As W content increases from 20% to 80%, the compression failure strain decreases from 1.43 to 1.34 with an amplitude of 6.2%, while the tension failure strain decreases from 0.3 to 0.036 with an amplitude of 88%.
(3) The materials show an obvious strain rate strengthening effect and thermal softening effect under dynamic compression. However, the dynamic compression strengths and strain rate sensitivities of the materials with different W contents show no obvious difference. For the materials with a W content of 50%, the dynamic compression strength improves from 60.2 MPa to 105.6 MPa as the strain rate increases from 4971 s^−1^ to 8753 s^−1^ at ambient temperature; meanwhile, it decreases from 64.3 MPa to 41.3 MPa as the material temperature increases from 25 °C to 200 °C at the strain rate of 5500 s^−1^.
(4) The Johnson–Cook constitutive (*A*, *B*, *n*, *C* and *m*) and failure parameters (*D*_1_~*D*_5_) were well-determined and predicted stress–strain curves are in good agreement with the experimental results. The results of this research would prove beneficial to the numerical studies, design and application of reactive materials.

## Figures and Tables

**Table 1:** Compositions and densities of the Al-PTFE-W reactive materials.

Material Types	Component Mass Ratios (wt. %)			TMD (g·cm^−3^)	Actual Density (g·cm^−3^)
	Al	PTFE	W		
M20	21.2	58.8	20	2.78	2.65
M50	13.2	36.8	50	4.09	3.97
M80	5.3	14.7	80	7.8	7.66

**Table 2:** Characteristic parameters of the Al-PTFE-W materials under quasi-static condition.

	Material Type	Elastic Modulus (MPa)	Yield Strength (MPa)	Ultimate Strength (MPa)	Critical Failure Strain
Compression	M20	508.5	10.6	22.1	1.43
	M50	628.8	11.4	19.1	1.38
	M80	734.6	16.5	20.9	1.34
Tension	M20	806	12.2	18.4	0.3
	M50	831	13.2	16.7	0.22
	M80	874	13.4	14.1	0.036

**Table 3:** Characteristic parameters of the Al-PTFE-W materials at elevated strain rates.

Material Type	Strain Rate (s^−1^)	Yield Strength (MPa)	Ultimate Strength (MPa)	Critical Failure Strain
M20	5560	28.5	67.3	0.42
	5983	28.7	78.9	0.49
	6862	30.8	100.3	0.52
	8160	34.4	98.7	0.63
	8730	32.6	105.9	0.67
M50	4971	27.9	60.2	0.36
	5556	29.6	64.3	0.41
	6023	27.9	68.3	0.5
	7326	30.7	85.9	0.56
	7996	36.5	90.2	0.57
	8753	34.8	105.6	0.6
M80	5222	41.3	65.4	0.38
	6340	46.7	72.3	0.48
	7143	41.8	77.2	0.61
	7912	48.9	102.6	0.66

**Table 4:** Characteristic parameters of the Al-PTFE-W materials at elevated temperatures.

Material Type	Temperature (°C)	Strain Rate (s^−1^)	Yield Strength (MPa)	Ultimate Strength (MPa)	Critical Failure Strain
M20	100	5500	27.3	51.9	0.37
	150		20.8	48.3	0.33
	200		23.1	47.6	0.4
M50	100	5500	28.3	62.1	0.41
	150		22.5	54.9	0.39
	200		23.3	41.3	0.34
M80	100	5200	35.7	62.9	0.32
	150		29.2	58.7	0.31
	200		36.6	51.5	0.31

**Table 5:** Johnson–Cook constitutive parameters of the materials.

Material Type	*A* (MPa)	*B* (MPa)	*n*	*C*	*m*
M20	10.6	45.9	0.81	0.062	1.19
M50	11.4	41.6	0.89	0.074	1.16
M80	16.5	24.2	0.43	0.067	1.32

**Table 6:** Johnson–Cook failure parameters of the materials.

Material Type	*D*_1_	*D*_2_	*D*_3_	*D*_4_	*D*_5_
M20	0.02	0.807	−1.873	−0.0455	−0.488
M50	0.043	0.731	−2.061	−0.0461	−0.399
M80	0.049	0.664	−2.3	−0.0459	−0.4

## Data Availability

Not applicable.
